# Supplementary material for: Marital rape and its impact on the mental health of women in India: A systematic review
Source: PLOS Glob Public Health. 2022 Jun 21;2(6):e0000601. doi: 10.1371/journal.pgph.0000601 (PMC10021972; doi:10.1371/journal.pgph.0000601)
Supplement: S1 File — (DOCX) [file pgph.0000601.s002.docx]

**Concept: Marital rape and domestic abuse in India**

**PubMed Search Strategy**

○ MeSH terms / subheadings

■ "Domestic Violence"[Mesh]

■ "Intimate Partner Violence"[Mesh]

■ "Battered Women"[Mesh]

■ "Rape"[Mesh]

■ "India"[Mesh]

○ Additional free text terms

■ "domestic violence"[tw] OR "partner violence"[tw] OR “spouse

violence”[tw] OR “spousal violence”[tw] OR “wife violence”[tw] OR

"domestic abuse"[tw] OR "partner abuse"[tw] OR “spouse

abuse”[tw] OR “spousal abuse”[tw] OR “wife abuse”[tw] OR

1

"domestic assault"[tw] OR "partner assault"[tw] OR “spouse

assault”[tw] OR “spousal assault”[tw] OR “wife assault”[tw] OR

"domestic battery"[tw] OR "partner battery"[tw] OR “spouse

battery”[tw] OR “spousal battery”[tw] OR “wife battery”[tw] OR

“abusive relationship”[tw] OR “abusive relationships”[tw] OR “violent

relationship”[tw] OR “violent relationships”[tw] OR "battered

woman"[tw] OR "battered women"[tw] OR "abused woman"[tw] OR

"abused women"[tw] OR “battered wife”[tw] OR “battered wives”[tw]

OR “rape”[tw] OR “rapes”[tw] OR “raped”[tw] OR “raping” OR

“sexual assault”[tw] OR “sexual assaults”[tw] OR “sexually

assaulted”[tw] OR “sexually assaulting”[tw]

■ “india”[tw] OR “indian”[tw] OR “indians”[tw] OR “south asia”[tw] OR

“south asian”[tw] OR “south asians”[tw] OR “southern asia”[tw] OR

“southern asian”[tw] OR “southern asians”[tw]

● Search string (Superset)

○ ("Domestic Violence"[Mesh] OR "Intimate Partner Violence"[Mesh] OR

"Battered Women"[Mesh] OR "Rape"[Mesh] OR "domestic violence"[tw]

OR "partner violence"[tw] OR “spouse violence”[tw] OR “spousal

violence”[tw] OR “wife violence”[tw] OR "domestic abuse"[tw] OR "partner

abuse"[tw] OR “spouse abuse”[tw] OR “spousal abuse”[tw] OR “wife

abuse”[tw] OR "domestic assault"[tw] OR "partner assault"[tw] OR “spouse

assault”[tw] OR “spousal assault”[tw] OR “wife assault”[tw] OR "domestic

battery"[tw] OR "partner battery"[tw] OR “spouse battery”[tw] OR “spousal

battery”[tw] OR “wife battery”[tw] OR “abusive relationship”[tw] OR

“abusive relationships”[tw] OR “violent relationship”[tw] OR “violent

relationships”[tw] OR "battered woman"[tw] OR "battered women"[tw] OR

"abused woman"[tw] OR "abused women"[tw] OR “battered wife”[tw] OR

“battered wives”[tw] OR “rape”[tw] OR “rapes”[tw] OR “raped”[tw] OR

“raping” OR “sexual assault”[tw] OR “sexual assaults”[tw] OR “sexually

assaulted”[tw] OR “sexually assaulting”[tw]) AND ("India"[Mesh] OR

“india”[tw] OR “indian”[tw] OR “indians”[tw] OR “south asia”[tw] OR “south

asian”[tw] OR “south asians”[tw] OR “southern asia”[tw] OR “southern

asian”[tw] OR “southern asians”[tw])

■ Results: 1,075
