# Supplementary material for: Marital rape and its impact on the mental health of women in India: A systematic review
Source: PLOS Glob Public Health. 2022 Jun 21;2(6):e0000601. doi: 10.1371/journal.pgph.0000601 (PMC10021972; doi:10.1371/journal.pgph.0000601)
Supplement: Attachment — Submitted filename: Response to reviewers.pdf [file pgph.0000601.s003.pdf]

13 March, 2022
Dr. Nafisa Halim
PLOS Global Public Health

Dear Dr. Halim,

Thank you for considering our manuscript, titled ‘Marital Rape and its impact on the Mental Health of women in India: A Systematic Review’ for publication in PLOS Global Public Health.

We would like to thank the reviewer, Dr. Hossain, for the careful and thorough reading of this manuscript, as well as thoughtful comments and suggestions, which will help in improving this paper. The table below details our responses.

We sincerely hope that the revisions we made to address these helpful comments are satisfactory. We are, of course, happy to make any further changes. Thank you for your kind consideration of our revised manuscript.

**Reviewer #1**	**Responses**
This article is a well-written systematic review on a critical psychosocial problem with profound implications on public health and social justice. The authors have outlined the overall scenario of marital rape and associated mental health outcomes in India.	Many thanks for your kind comment. We really appreciate it.
First, some definitions of marital rape should be cited and discussed in the background. This is critical because of the lack of any specific definition that is used in India. It is okay to cite global literature to inform the readers about the definitions used elsewhere, acknowledging a lack of health, social, administrative, and legal definitions in that context.	We have now added another definition and cited the same in the introduction (see lines 54-61, pg. 4).
Second, the timeframe of the literature search should be stated as "We searched literature from X date to Y date" where X date means the beginning of publications, not the date of searching the same. However, "Y date" can be the date of searching for the last time, which shows the time limit of the included literature. If	We agree with the reviewer on the need to be more specific. Thank you for noting this gap. We have updated the timeline.<br>(See line 103, pg. 6)

"Y date" has two values, for example, the authors searched once and repeated the same later, that should be stated clearly, without affecting the beginning date (X date of the search timeframe).	
Third, the abstract mentions the "Indian sub-continent" to define the geographic focus, whereas the eligibility criteria focus on Indian women only. It is recommended that the authors should use the study population consistently.	This is an important point. We have now corrected the text, changing Indian subcontinent to “India”.<br>(See lines 23, 30, pg. 2)
Fourth, although estimating the prevalence of marital rape was part of the objective, the authors did not provide details on this topic in the results section. It should be described narratively in one-two paragraphs alongside specific examples or explanations from the included studies. It should be reflected in the abstract as well.	We have now illustrated the prevalence of marital rape in the results section, both in the body of the manuscript (see lines 216-224, pg. 17) and the abstract (see lines 37-38, pg. 2).
Fifth, the authors may wish to update their tables mentioning key information on the study methods for each recruited study. These should include but are not limited to information on recruitment strategy, sampling strategy, study design, and analytical approach. It will help the readers to understand the strengths and weaknesses of the included studies.	The tables have been expanded to provide a more comprehensive study description.<br><br>Table 1a: Included columns Sample type and size, Study design, Sampling strategy, Recruitment strategy (see table 1a, pg. 8-9).<br>Table 1b: Included columns Study population, Study design, Sampling strategy and sample size, Recruitment Strategy (see table 1b, pg. 9-10)<br><br>Table 2a: Included columns Spousal violence and Statistical measures (see table 2a, pg. 10-14).<br>Table 2b: Included column Statistical measures (see table 2b, pg. 14-15)
Sixth, the authors may specify and report whether this study focuses on specific mental health outcomes or all mental health outcomes. The included studies have many mental health issues reported in respective samples. In this scenario, the authors may focus on specific	Thank you for this point, we are focusing on DSM syndromes that are plausibly related to marital rape, and also reporting secondary outcomes that arose in our identification of those studies. We have edited the manuscript to ensure that the study primary and secondary outcomes of are clear. The

disorders recognized in DSM or ICD or include any psychological condition associated with marital rape, making the review more inclusive from an evidence synthesis perspective.	outcome measures including primary outcomes of PTSD and depression, and the secondary outcome of suicidality are illustrated in the abstract (see lines 33-35, pg. 2) and the body of the manuscript (see lines 135-138 and lines 150-151, pg. 7; lines 227-230, pg. 17-18).
Lastly, the discussion should present some insights or recommendations for future policymaking and practice. Marital rape is a critical social problem that should be addressed through multilevel efforts, and this study can use the synthesized evidence to provide some recommendations that the authors find suitable in the context of India.	Although the paper aimed to drive research more so than policy, we agree with the reviewer that given the importance of the issue, our analysis can be used as the base to move the discourse and actions forward. We have now added some recommendations in the discussion section (see lines 381-385, pg. 23-24).
